# HMGA1 is a crucial mediator of colon tumorigenesis driven by the loss of APC

**DOI:** 10.1172/JCI187442

**Published:** 2025-02-03

**Authors:** Yuxiang Wang, Mikayla Ybarra, Zhenghe Wang

**Affiliations:** 1 Department of Genetics and Genome Sciences and; 2 Case Comprehensive Cancer Center, Case Western Reserve University, Cleveland, Ohio, USA.

## Abstract

Colorectal cancer is the second leading cause of cancer death in the United States. The adenomatous polyposis coli (APC) pathway plays a critical role in colorectal tumorigenesis, but the mechanism is not fully understood. In this issue of the *JCI*, Luo and colleagues used genetically engineered mouse models to show that high mobility group A (HMGA1) is a critical mediator in the development of colon tumors driven by the loss of the *Apc* gene. HMGA1 activated the transcription of Achaete-Scute Family BHLH Transcription Factor 2 (ASCL2), which regulated intestinal stemness and promoted colon tumorigenesis.

## Mouse models for colorectal cancer

Colorectal cancer is the second leading cause of cancer death in the United States, with 153,000 new cases and 53,000 deaths projected in the US in 2024 (1). Colorectal cancer development is driven by a series of genetic mutations, such as those in *APC*, *KRAS*, *TP53*, and *PIK3CA*, among others. (2). *APC* is mutated at a very early stage and results in benign adenoma formation (2). The WT APC is required for a complex with axin that eventually leads to β-catenin’s phosphorylation by GSK3β and its subsequent degradation (2). When APC is inactivated by mutations, β-catenin is accumulated and translocated into the nucleus, where it interacts with the transcription factor TCF-4, and they both transcriptionally activate a variety of genes, especially those involved with cellular proliferation and survival (2). With the accumulation of other mutations in *KRAS*, *PIK3CA*, and *TP53*, the benign adenomas become malignant adenocarcinoma and eventually metastasize (2). Historically, *Apc* multiple intestinal neoplasia (*Apc*^Min^) mice (3), which harbor an *Apc* nonsense mutation (4), are widely used to model colorectal tumorigenesis. However, most tumors developed in the *Apc*^Min^ mice only occur in the small intestines. While colon tumors in the context of *Apc*^Min^ can be induced using the bacterium enterotoxigenic Bacteroides fragilis (ETBF), it is unclear whether ETBF plays a causal role in human colorectal tumorigenesis. To circumvent this problem, Eric Fearon’s group developed a *CDX2P-CreER^T2^* transgenic mouse strain that conditionally deletes genes in the colon. Notably, the *CDX2P-CreER^T2^Apc^fl/fl^* mice develop adenomas in the colon, not in the small intestine (5).

## HMGA in colon tumorigenesis

Many genes, which are expressed in embryonic development and adult stem cells but inactivated in differentiated tissues, are reactivated in cancer cells. High mobility group A (*HMGA1*) is one of those genes. High mobility group (HMG) proteins, of which HMGA1 is a member, are the second-most abundant proteins in chromosomes, orchestrating chromatin remodeling processes and governing gene transcription in eukaryotic cells (6). In many cancer types, including colorectal cancer, HMGA1 is overexpressed (6). Further, APC loss promotes HMGA1 expression through the mobilization of β-catenin/TCF-4 transcriptional complex (7).

In this issue of the *JCI*, Luo et al. demonstrated that HMGA1 plays an important role in colon tumorigenesis (8). First, using the *CDX2P-CreER^T2^Apc^fl/fl^* mouse model, the authors showed that knockout of *Hmga1* in the mice reduced tumor burden and extended their survival (8). Second, *Hmga1* knockout in the context of *Apc^Min^* with ETBF, reduced tumor number, regardless of whether the HMGA1 deficiency was global or specific to the intestinal epithelium (8). Notably, even heterozygous *Hmga1* knockout reduced colon tumorigenesis in both mouse models (8).

The epithelia of the colon and small intestine are constantly renewed by the intestinal stem cells in crypts (9). In the small intestine, a pool of proliferative crypt-base columnar (CBC) cells that are marked by LGR5 is generally believed to be the intestinal stem cells (9). The LGR5^+^ stem cells divide every 24 hours and generate transit-amplifying cells (9), which migrate upward and differentiate into absorptive enterocytes, enteroendocrine cells, and goblet cells (9). These differentiated cells form the finger-like structure of the villus. Paneth cells escape this upward flow, migrate to the bottom of the crypt, and intermingle with LGR5^+^ stem cells (9). Notably, most recent studies showed that the stemness potential is also found in the intestinal crypt isthmus, which participates in intestinal homeostasis and regeneration (10, 11). Although the colon does not have villi, the crypt structure of the colon is similar to that of the small intestine. Notably, paneth cells are not detected in the colon, but it has been suggested that paneth-like cells are intermingled with the LGR5^+^ stem cells at the bottom of crypts. To interrogate how HMGA1 affects the cell composition in the colon, Luo and authors performed scRNA-seq in proximal colon crypt cells from *CDX2P-CreER^T2^Apc^fl/fl^ Hmga1^+/+^* and *CDX2P-CreER^T2^Apc^fl/fl^ Hmga1^–/–^* mice. Given the role of HMGA1 in adult stem cells, it is not surprising that stem cell populations were decreased in the *Hmga1*-knockout colon crypts compared with those of *Hmga1* WT mice (8). Paneth-like cells were also decreased in knockout colon crypts, which is consistent with a previous study by the same group showing that HMGA1 induces SOX9 gene expression, thereby promoting Paneth-like cell differentiation (12). However, the relevance of the Paneth-like cell in colon tumorigenesis is not clear. Luo and authors claimed that HMGA1 depletion had minimal effects on colon epithelial regeneration under homeostatic conditions, based on the observation that *Hmga1* heterozygous–knockout mice had normal development and lifespans (8). This claim needs to be solidified by careful examination of intestines in mice with intestinal-specific knockout of *Hmga1* at young and old ages.

**Figure 1:**
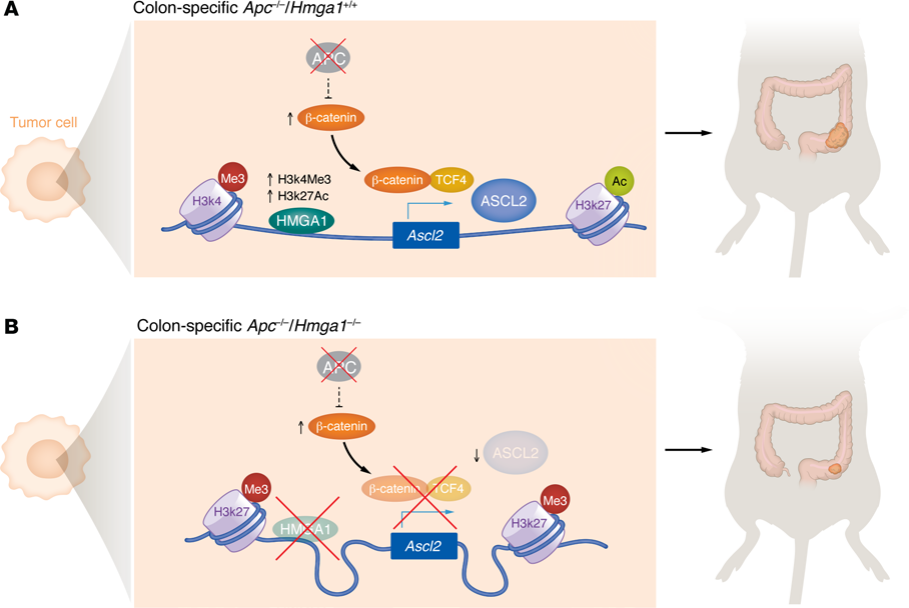
HMGA1 plays a pivotal role in colon tumorigenesis driven by the loss of APC. (**A**) APC loss results in β-catenin stabilization and nuclear translocation, thereby activating its target gene expression, including *Ascl2*. HMGA1 directly binds to the *Ascl2* promotor, upregulates activating histone markers (H3K4me3 and H3K27Ac), decreases the repressive histone marker (H3K27me3), increases chromatin accessibility, and facilitates β-catenin–mediated expression of ASCL2. (**B**) In the context of APC loss, HMGA1 deficiency reduces ASCL2 expression and decreases colon tumorigenesis.

Trajectory and cell state analyses showed that HMGA1 promotes an undifferentiated stem cell state. To identify the underlying molecular mechanisms by which HMGA1 maintains the intestinal stem cell state and promotes colon tumorigenesis, Luo and authors focused on ASCL2 based on their observations that ASCL2 positively correlated with HMGA1 expression in both mouse crypt cells and human colorectal cancer samples (8). The ASCL2 transcription factor acts as a Wnt-responsive switch to control stemness in the intestine (13). Luo and colleagues demonstrated that HMGA1 is directly bound to the ASCL2 promoter and activated ASCL2 expression in human colon cancer cells (Figure 1). Furthermore, overexpression of ASCL2 in HMGA1-knockdown CRC cells partially rescued the slow growth of the *HMGA1*-silenced cells. HMGA1 activated ASCL2 gene expression by increasing the activating histone marks (H3K4me3, H3K27Ac) and decreasing the repressive histone marker (H3K27me3), thereby opening the *ASCL2* promotor region (8). However, how HMGA1 promotes the deposition of activating histone marks remains to be determined.

Besides the epithelia, lamina propria is the connective tissue layer containing blood vessels, lymphatics, and immune cells (14). Interestingly, the scRNA-seq analysis indicated that the quantity of CD4^+^ and CD8^+^ T cells was increased in the *CDX2P-CreER^T2^Apc^fl/fl^ Hmga1^–/–^* colon crypt cells compared with the WT *Hmga1* counterpart (8). However, it remains to be determined if the increased T cell infiltration is caused by the loss of *Hmga1* in the epithelial cells or T cells. Nonetheless, the gene signature enrichment analysis in the epithelial cells showed that the INF-α and INF-γ pathways were enriched, suggesting that loss of HMGA1 in the epithelial cells may affect the immune microenvironment in the colon and result in the increased T cell infiltration (8). The exact mechanisms warrant further investigation.

## Conclusion

Luo and authors provided compelling evidence that HMGA1 plays a pivotal role in colon tumorigenesis driven by the loss of APC through regulating ASCL2 to promote an undifferentiated stem cell state (8). HMGA1 increases the accessibility of transcription factor ASCL2, a crucial regulator of stemness, thereby activating its transcription. This study suggests that HMGA1 is an epigenetic gatekeeper for the APC/β-catenin–regulated transcriptional network.

## Version 1 02/03/2025

Electronic publication
